# Elderly patients with non-specific complaints at the emergency department have a high risk for admission and 30-days mortality

**DOI:** 10.1186/s12877-023-04621-7

**Published:** 2024-01-03

**Authors:** Karin Erwander, Kjell Ivarsson, Mona Landin Olsson, Björn Agvall

**Affiliations:** 1 Department of Clinical Sciences, Lund University, Lund, Sweden; 2 Department of Research and Development, Region Halland, Halmstad, Sweden

**Keywords:** Non-specific complaint, Atypical presentation, Elderly patients, Emergency department, Geriatric emergency medicine, Admissions

## Abstract

**Background:**

Older adults have complex medical needs that causes increased use of resources at the emergency department (ED). The prevalence of non-specific complaint (NSC) as a chief-complaint in the ED is common among older adults and is not prioritized even though possibly having worse clinical outcome. The objective was to study hospital admission and mortality for older adults visiting the ED with NSC compared to specific complaints such as dyspnea, chest pain and abdominal pain.

**Methods:**

A retrospective observational study of older adults visiting the ED with NSC and specific complaints; dyspnea, chest pain and abdominal pain was performed. Chief-complaint were collected from electronic medical records. Fatigue, confusion, non-specific complaints, generalized weakness and risk of falling were defined as non-specific complaint (NSC) when registered as chief-complaint at the ED. Admission rate and 30-days mortality were the primary outcomes.

**Results:**

A total of 4927 patients were included in the study based on chief-complaint; patients with chest pain 1599 (32%), dyspnea 1343 (27%), abdominal pain 1460 (30%) and NSC 525 (11%). Patients with dyspnea and NSC had the highest hospital admission rate 79% vs 70% compared to patients with chest pain (63%) and abdominal pain (61%) (*p* =  < 0.001). Patients with NSC had a mean LOS 4.7 h at the ED which was significantly higher compared to chest pain, dyspnea and abdominal pain. Mean bed-days for the whole population was 4.2 days compared to patients with NSC who had a mean LOS of 5.6 days. NSC and dyspnea were both associated with the highest 30-day mortality.

**Conclusion:**

Older patients who present with NSC at the ED are associated with a high risk for admission and 30-days mortality. In addition, patients with NSC have a longer LOS at the ED, a high admission rate and the highest number of bed-days once admitted. This study indicates that ED staff should be more vigilant when an elderly patient presents with NSC at the ED. Further studies and guidelines are needed to improve the management of these individuals.

**Supplementary Information:**

The online version contains supplementary material available at 10.1186/s12877-023-04621-7.

## Background

Chest pain, dyspnea and abdominal pain are all specified symptoms frequently appearing at the Emergency Department (ED) that can be caused by a limited number of possible diagnoses. In those patients having these specific chief-complaints, there are usually protocols and work-up programs that can be followed and support healthcare decisions [1–3]. However, there are also non-specific complaints (NSC) that appear at the ED such as fatigue, generalized weakness, altered mental status, failure to eat and drink, reduced mobility and falling where ED protocols usually do not apply [4, 5]. Studies have shown that NSC are frequent among patients at the ED and highest among older adults [4, 6]. Older adults often have multiple co-morbidities which can disguise classical signs and symptoms as well as age-related physiological changes such as failure to develop fever and lack of chest pain when having a heart attack which may result in NSC in a situation when deteriorating [7, 8].

Older adults have more complex medical needs that consequently results in longer waiting time at the ED and increased use of resources [9–13]. The risk of adverse events for elderly patients increases with length of stay (LOS) at the ED [14, 15]. Older patients with NSC often appear in the ED, and even though this can mean worse clinical outcome and longer LOS, they are still a patient category that is usually not prioritized [16–21]. NSC are one of the most challenging conditions for an ED-physician since there are not any specific protocols to follow and the cause of NSC can be caused by everything from life threatening conditions, lack of home health care or natural aging [4, 20, 22].

The aim of this study was to compare older adults with NSC at the ED with patients with specific chief-complaints such as dyspnea, chest pain and abdominal pain regarding admission rate and 30-day mortality.

## Methods

### Setting and design

This retrospective observational study was conducted with data of older adults ≥ 65 years of age visiting one of the ED in Region Halland (RH) located on the southwest of Sweden. Within RH, there are three acute care hospitals, 40 inpatient wards, two emergency departments and 30 outpatient specialized clinics. The study period was between 1st January 2016 and 31st December 2016. During 2016, a total of 314,784 individuals lived in RH. The proportion of individuals aged ≥ 65 years were 71,688 (23%). The data collection was generated from the Regional Healthcare Information Platform (RHIP) provided of RH. RHIP database that contains all information from primary and secondary healthcare including all electronic medical records, assessment instruments and examinations [23]. All patients visiting the ED are triaged according to Rapid Emergency Triage and Treatment System (RETTS) and chief-complaint are registered data were retrieved from RHIP [24].

### Patients and selection

**Fig. 1:**
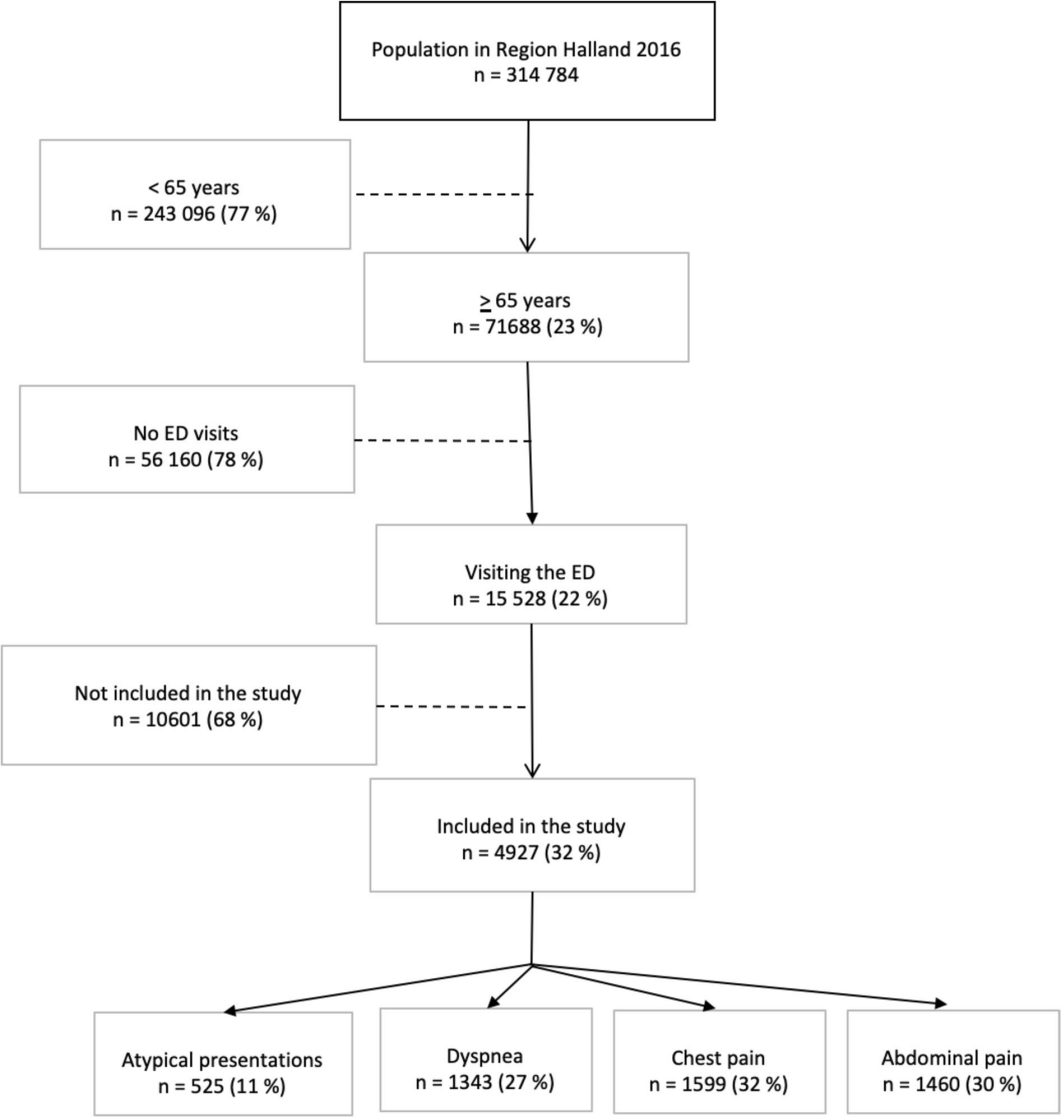
Flow chart inclusion in the study. Note: n = number, ED = emergency department, NSC = non-specific complaint

A total of 15,528 (22%) individuals aged ≥ 65 years visited the ED in RH at least one time during 2016. In the study, there were 4927 individuals who were ≥ 65 years of age visiting the ED with NSC, dyspnea, chest pain or abdominal pain. For patients with multiple visits to the ED only the first visit during the study period was registered to minimize data deviations. Trauma was the number one reason for ED-visit but was excluded in this study based on the broad variety in symptoms and severity level. Dyspnea, chest pain and abdominal pain were then selected based on being the top three reasons for visit. Included in NSC were the terms fatigue, altered mental status, falling, generalized weakness and non-specific complaints. Figure 1 shows a flow chart of inclusion in the study.

### Data collection

The data variables extracted were age, gender, comorbidities before visits to the ED, chief-complaint for ED-visit, LOS at ED, admission rate, in-hospital LOS and 30-days mortality. Comorbidities that each included patient had before their first ED-visit during 2016 were collected. All diagnoses were registered according to the International Classification of Disease-10 (ICD-10). The most common diagnosis´ were then categorized into the following groups; hypertension, ischemic heart disease (IHD), atrial fibrillation (AF), heart failure (HF), cerebrovascular insult (CVI), diabetes mellitus (DM), chronic obstructive pulmonary disease (COPD), musculoskeletal pain, psychiatric disorders and malignancy as described in Appendix 1. Patients with none of the above listed diagnosis were categorized as previously healthy. Patients were also categorized according to their health status if they were previously healthy, had diagnosis from 1–3 diagnostic groups or diagnosis from ≥ 4 diagnostic groups.

### Definitions

The chief-complaint for ED-visits were collected from electronic medical records. Fatigue, confusion, non-specific complaints, generalized weakness and risk of falling was defined as NSC when registered as chief-complaint at the ED visit. All other complaints were classified as specific complaints.

### Outcome measures

The primary outcome measures were hospital admissions and 30 days mortality. The secondary outcome measure was LOS at ED, demographic characteristics, previous health condition for the older adults that visits the ED and in-hospital LOS.

### Statistical analysis

Descriptive statistics were used to characterize patients’ demographics. Continuous variables were described as means + standard deviation (SD) and analyzed using Student’s t-test and One-way-ANOVA. Categorical variables were analyzed using Chi-2 tests and summarized using frequency and percentages.

According to the WHO classification, patients ≥ 65 years of age were classified as older adults and then categorized into three different groups; 65–74 years, 75–84 years and ≥ 85 years [25]. The number of diagnostic groups was registered for each individual patient and based on this, the patients were categorized according to their health status if they were previously healthy, had diagnosis from 1–3 diagnostic groups or diagnosis from ≥ 4 diagnostic groups. LOS at the ED were categorized as < 4 h or > 4 h [26].

Multivariate regression analysis for hospital admission were performed adjusted for the complaints; NSC, dyspnea, chest pain and abdominal pain. The risk for hospital admission was analyzed overall and separated according to age-groups, comorbidities and time at ED. The age-groups were 65–74 years, 75–84 years and ≥ 85 years. The comorbidities were grouped as previously healthy, 1–3 and ≥ 4 comorbidities. The time at ED was grouped as < 4 or > 4 h at ED. There was a Cox regression analysis for 30 days mortality for NSC, dyspnea, chest pain and abdominal pain that were adjusted for gender, age, comorbidities, time at ED, revisit within 72 h from first ED visit, hospital admission at index, bed days > 6 days when admitted and having readmission within 30 days. There were no missing values in the data collection.

A *p*-value < 0.05 was considered statistically significant. The analyses were performed with IBM SPSS Statistics 27, Armonk, New York, USA.

## Results

A total of 15,528 patients aged ≥ 65 years visited the ED in RH during 2016. Of these, 4927 (32%) were included in the study based on chief-complaint. The top 10 reasons for ED-visits are shown in Table 1. The distribution of chief-complaints was 1599 (32%) patients with chest pain, 1343 (27%) with dyspnea, 1460 (30%) with abdominal pain and 525 (11%) with NSC.

**Table 1:** Top 10 reasons for ED-visit in RH during the year of 2016

1	Trauma	2574 (21)
2	Chest pain	1599 (13)
3	Abdominal pain	1460 (12)
4	Dyspnea	1343 (11)
5	Musculoskeletal pain	1130 (9)
6	Infection	1101 (9)
7	Neurology	1011 (8)
8	Arrhythmia	849 (7)
9	Vertigo	649 (5)
10	NSC	525 (4)

*NSC* non-specific complaint. Percentages are shown in parentheses

Descriptive statistics of demographic data are shown in Table 2. Patients with NSC had a mean age of 80 years. The patients who sought for dyspnea had a mean age of 80 years, chest pains 77 years and abdominal pain 78 years (*p* = 0.001). Patients with NSC had a mean LOS of 4,7 h at the ED which was significantly higher compared to chest pain, dyspnea and abdominal pain. Mean bed-days for the whole population was 4.2 days compared to patients with NSC who had a mean LOS of 5.6 days.

**Table 2:** Illustrate demography of the study population that made visits to the ED during 2016 with NSC, dyspnea, chest pain and abdominal pain. And the association between type of complaint and clinical outcome

	**Total**	**NSC**	**Dyspnea**	**Chest pain**	**Abdominal pain**	***p*-value**
Total	4927	525 (11)	1343 (27)	1599 (32)	1460 (30)	< 0.001
Age, mean (SD)	78 (8.1)	80 (8.3)	80 (8.3)	77 (7.8)	78 (7.9)	< 0.001
65–74, n (%)	1941 (39)	168 (32)	427 (32)	715 (45)	631 (43)	< 0.001
75–84, n (%)	1865 (38)	210 (40)	513 (38)	586 (37)	556 (38)	
≥ 85, n (%)	1121 (23)	147 (28)	403 (30)	298 (19)	273 (19)	
Gender						
Female, n (%)	2394 (49)	262 (50)	639 (48)	816 (51)	677 (46)	0.06
Male, (%)	2533 (51)	263 (50)	704 (52)	783 (49)	783 (54)	
Previous health condition						
Healthy, n (%)	1486 (30)	158 (30)	306 (23)	516 (32)	506 (35)	< 0.001
1–3 diagnostic groups, n (%)	2800 (57)	316 (60)	775 (58)	862 (54)	847 (58)	
≥ 4 diagnostic groups, n (%)	641 (13)	51 (10)	262 (20)	221 (14)	107 (7)	
Duration ED, mean (SD)	4.3 (2,3)	4.7 (2.5)	4.3 (2.4)	4 (2.2)	4.5 (2.3)	< 0.001
Duration ED > 4 h, n (%)	2422 (49)	299 (57)	635 (47)	734 (46)	754 (52)	< 0.001
Admission, n (%)	3321 (67)	368 (70)	1060 (79)	1003 (63)	890 (61)	< 0.001
Re-visit 72 h, n (%)	214 (4)	25 (5)	42 (3)	36 (2)	111 (8)	< 0.001
Bed-days, mean (SD)	4.2 (6.0)	5.6 (8.3)	5.4 (7.2)	3.6 (6.2)	3.2 (6.0)	< 0.001
30-days mortality, n (%)	272 (6)	45 (9)	128 (10)	32 (2)	67 (5)	< 0.001

*NSC* non-specific complaint, *n* number, *SD* standard deviation, *NSC* non-specific complaint, *ED* emergency department

Patients with dyspnea and NSC had the highest hospital admission rate 79% vs 70% compared to patients with chest pain (63%) and abdominal pain (61%) (*p* =  < 0.001) which is shown in Table 2.

The overall 30-days mortality in the total study group was 6% where patients with dyspnea had the highest mortality rate of 10% followed by patients with NSC 9%, abdominal pain 5% and chest pain 2% which is displayed in Table 2.

A multivariate regression analysis for hospital admission adjusted for age, previous health conditions and LOS at the ED had an Odds ratio 1.6 (Confidence Interval [CI] 1.27–2.00) for dyspnea compared to NSC. The analysis for hospital admission is also separated for age, previous health conditions and LOS at the ED, illustrated in Table 3.

**Table 3:** Risk of hospital admissions adjusted for each complaint regarding overall risk and separated by age groups, number of diagnoses/comorbidities and time at Emergency Department

**Chief complaint**	**Overall**											
	Odds ratio	Lower 95% C.I	Upper 95% C.I	*p*-value								
NSC	Ref.			< 0.001								
Dyspnea	1.60	1.27	2.00									
Chest pain	0.72	0.60	0.90									
Abdominal pain	0.67	0.54	0.83									
**Age-groups**	**65–74**				**75–84**				** > 84**			
	Odds ratio	Lower 95% C.I	Upper 95% C.I	*p*-value	Odds ratio	Lower 95% C.I	Upper 95% C.I	*p-*value	Odds ratio	Lower 95% C.I	Upper 95% C.I	*p-*value
NSC	Ref.			< 0.001	Ref.			< 0.001	Ref.			< 0.001
Dyspnea	1.55	1.07	2.25		1.21	0.83	1.76		2.82	1.73	4.59	
Chest pain	0.93	0.66	1.32		0.58	0.41	0.82		0.87	0.56	1.37	
Abdominal pain	0.80	0.56	1.12		0.51	0.36	0.73		1.15	0.72	1.83	
**Diagnostic groups**	**Previously healthy**				**1–3 diagnostic groups**				** ≥ 4 diagnostic groups**			
	Odds ratio	Lower 95% C.I	Upper 95% C.I	*p*-value	Odds ratio	Lower 95% C.I	Upper 95% C.I	*p*-value	Odds ratio	Lower 95% C.I	Upper 95% C.I	*p*-value
NSC	Ref.			< 0.001	Ref.			< 0.001	Ref.			0.001
Dyspnea	0.99	0.64	1.54		1.68	1.26	2.26		2.87	1.44	5.71	
Chest pain	0.53	0.36	0.79		0.75	0.57	0.98		1.17	0.61	2.27	
Abdominal pain	0.49	0.33	0.74		0.69	0.53	0.91		1.87	0.88	4.00	
**Time at ED**	** < 4 h ED-visit**				** > 4 h ED-visit**							
	Odds ratio	Lower 95% C.I	Upper 95% C.I	*p*-value	Odds ratio	Lower 95% C.I	Upper 95% C.I	*p*-value				
NSC	Ref.			< 0.001	Ref.			< 0.001				
Dyspnea	2.95	2.07	4.19		0.96	0.71	1.30					
Chest pain	1.19	0.87	1.63		0.43	0.32	0.58					
Abdominal pain	0.74	0.54	1.02		0.61	0.46	0.82					

*NSC* non-specific complaint, *ED* emergency department

Overall Cox regression for 30-days mortality with NSC as reference showed a Hazard ratio of 1.12 (CI 0.80–1.58) for dyspnea, 0.23 (CI 0.15–0.36) and 0.53 (CI 0.36–0.77) for chest pain and abdominal pain respectively. Table 4 shows an adjusted Cox regression model and Hazard ratio for NSC, dyspnea, chest pain and abdominal pain.

**Table 4:**
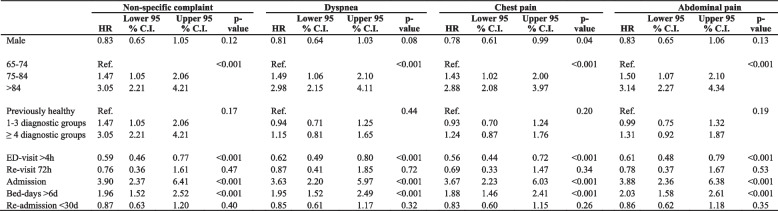
Cox-regression 30-days mortality

*NSC* non-specific complaint, *ED* Emergency Department

## Discussion

Among older adults visiting the ED, those with NSC and dyspnea had the highest age. NSC as the chief-complaint was associated with a higher rate of ED duration > 4 h and readmission within 72 h. Patients with NSC had the longest in-hospital LOS. Older adults with dyspnea in the ED were associated with the highest hospital admission rate followed by NSC which had a higher hospital admission rate compared to patients presenting with chest and abdominal pain. Earlier studies have shown a similar high admission rate for elderly patients with chief complaint dyspnea and chest pain [27]. The high admission rate for geriatric patients may be a sign of the difficulties to distinguish between life-threatening conditions or the sign of chronic geriatric diseases at the ED. The 30-day mortality rate for patients with dyspnea and NSC were significantly higher compared to patients having chest pain or abdominal pain at the ED.

The present study has shown that patients in the ED with NSC have a high average age, which is consistent with other studies [4–6]. Increasing age entails an increased risk of hospital admission and likewise the occurrence of ≥ 4 comorbidities. LOS at ED ≥ 4 h was associated with a reduced risk of hospitalization which may seem odd. It was likely a certain degree of selection and that long LOS in the ED could be used as an observation time in practice.

In this study, 11% of the study cohort were defined as having NSC as their chief-complaint and in comparison, previous studies have shown that up to 20% of older adults at the ED present with NSC [28]. The difference may be due to the lack of international definition of non-specific complaints. Most commonly fatigue, generalized weakness, altered mental status, failure to eat and drink, reduced mobility and falling are considered NSC but sometimes these symptoms are referred to as the geriatric syndrome at the ED [20]. Patients with NSC have a poor clinical outcome and are often prioritized as low acuity at the ED. Studies show that screening for frailty could be one useful parameter to improve triage for older adults at the ED. Frailty appears to be of more use than chronological age when predicting outcome for geriatric trauma patients [29, 30]. Several studies have shown that a geriatric approach at the ED will shorten LOS at the ED and in-hospital LOS once admitted [31, 32].

The chief complaint with the highest risk for hospital admission was dyspnea followed by NSC. Still, NSC was associated with higher admission risk compared to both chest and abdominal pain and this finding was regardless of age group, degree of comorbidity and time at ED. Older patients with NSC were associated with a significantly higher 30-days mortality rate compared to patients with chest pain and abdominal pain and the mortality rate was comparable to patients with dyspnea, these data’s are comparable with previous studies [4, 33–35]. Chest pain as chief complaint will be prioritized higher at the ED compared to NSC. This study is in line with earlier studies and shows that patients with NSC have a higher admission rate and mortality rate than patients presenting with chest pain at the ED [36]. In addition, patients with NSC have a longer LOS at the ED, a high admission rate and the highest number of bed-days once admitted. Despite the lower number of patients with NSC in this study the 30-days mortality rate were in line with previous studies [4].

These results demonstrate that NSC in older adults can be difficult to assess for ED staff even though these individuals may be at significant risk for hospital requirements and 30-day mortality. There may be a need to improve routines regarding the handling of this patient group in the ED and previous study have reported there are limitations in existing risk stratification instruments for older adults visiting the ED [37].

## Conclusion

Older patients who present with NSC at the ED are associated with a high risk for hospital admission and 30-days mortality. In addition, patients with NSC compared to patients with SC have a longer LOS at the ED, a high admission rate and the highest number of bed-days once admitted. This study indicates that ED staff should be more vigilant when an elderly patient presents with NSC at the ED. Further research is needed to approach how to best care for older patients with NSC to reduce morbidity and mortality.

## Limitations

In the study, it is not possible to evaluate the degree of acuity or morbidity in terms other than the number of comorbidities. Nor can frailty be assessed, which is likely to be of decisive importance in this patient group. Trauma is a common diagnosis in the ED and was not included as a chief complaint in this study. The intention was to identify NSC and compare them with chief complaints that are judged to be relatively serious in the ED and where there are guidelines on how these patients should be managed at ED. Trauma has a wide variety of conditions and was not assessed being not appropriate in this regard.

The terminologies fatigue, altered mental status, falling, generalized weakness and non-specific complaints to define NSC could have been somewhat restrictive and leading to lower acceptance rate. However, these chief-complaint used compiled to a high rate compared to others and the purpose was not to overlap to specific complaint associated with specific diseases such as vertigo and neurology.

It should be emphasized that the results in this study are only associations, and it is not possible to draw any conclusions regarding causality.

### Supplementary Information

**Additional file 1: Appendix 1. **Describes diagnostic groups categorized according to the International Classification of Disease-10 (ICD-10).

## Data Availability

The datasets generated and/or analyzed during the current study are not publicly available due to the data is retrieved from patient’s hospital records which is included in the Swedish Health Care act which applies to Swedish secrecy act according to Swedish legislation. The data will be shared on reasonable request to the corresponding author.
