# Supplementary material for: Elderly patients with non-specific complaints at the emergency department have a high risk for admission and 30-days mortality
Source: BMC Geriatr. 2024 Jan 3;24:5. doi: 10.1186/s12877-023-04621-7 (PMC10762826; doi:10.1186/s12877-023-04621-7)
Supplement: Supplementary file 1 — Additional file 1: Appendix 1. Describes diagnostic groups categorized according to the International Classification of Disease-10 (ICD-10). [file 12877_2023_4621_MOESM1_ESM.pdf]

Appendix for “Elderly patients with non-specific complaints at the Emergency Department have a high risk for admission and 30-days mortality”

Appendix 1. Describes diagnostic groups categorized according to the International Classification of Disease-10 (ICD-10).

Diagnostic group	ICD-10
Hypertension	I10-I15
IHD	I20-I25
AF	I48
HF	I50
CVI	I60-I69
DM	E10-E14
COPD	J43-J45
Musculoskeletal pain	M05-M06, M15-M19, M54, M48, M79
Psychiatric disorders	F00-F03, F10-F48, R54, G30
Malignancy	C00-C97

Note; IHD = ischemic heart disease, AF = atrial fibrillation, HF = heart failure, CVI = cerebrovascular insult, DM = diabetes mellitus, COPD = chronic obstructive pulmonary disease.
